# Interdisciplinary therapy of extremity soft tissue sarcomas: current evidence on systemic treatments and the role of reconstructive surgery

**DOI:** 10.1515/iss-2025-0035

**Published:** 2025-11-11

**Authors:** Yonca Steubing, Christoph Wallner, Marcus Lehnhardt, Eren Celik, Christian Baues, Beate Schultheis, Anke Reinacher-Schick, Maria Füth, Jannik Hinzmann, Flemming Puscz, Patrick Stefan Harenberg, Felix Reinkemeier

**Affiliations:** Department of Plastic Surgery, Hand Surgery, Burn Center and Sarcoma Center, 39060 BG University Hospital Bergmannsheil Bochum, Bochum, Germany; Department of Radiation Oncology, Clinic of the Ruhr-University Bochum, Marien Hospital Herne, Herne, Germany; Department of Hematology, Oncology and Palliative Care, St. Josef-Hospital, Clinic of the Ruhr-University Bochum, Bochum, Germany

**Keywords:** soft tissue sarcoma, interdisciplinary therapy, radiotherapy, systemic therapy, oncology

## Abstract

**Objectives:**

Soft tissue sarcomas are rare and heterogenous tumors, that often pose diagnostic and therapeutic challenges. Guideline-compliant therapy includes surgical resection with negative margins, supplementary radiotherapy and, if necessary, accompanied by systemic therapy. Diagnostic and therapeutic approaches should be discussed and planned in an interdisciplinary oncological conference. The aim of this article is to provide an overview of guideline-based interdisciplinary therapeutic options, limitations and prospects. An exemplary case is used to illustrate interdisciplinary concepts.

**Case Presentation:**

A six-year-old girl with a locally advanced epithelioid sarcoma of the right hand was recommended to receive a hand amputation. Due to a request for limb-preservation, the patient presented at various sarcoma centers and initially received neoadjuvant radiotherapy. This resulted in a reduction of the tumor mass so that a tumor resection could subsequently be performed. Despite pronounced local findings, microscopically negative margins and a satisfactory postoperative result in hand function were achieved.

**Conclusions:**

This case highlights the critical role of interdisciplinary collaboration and centralized care in the treatment of soft tissue sarcomas. Plastic reconstructive surgery enables radical oncological resection while preserving function, especially in anatomically complex cases. Early referral to a specialized sarcoma center and adherence to current guidelines (e.g., ESMO-EURACAN-GENTURIS) are essential to achieving optimal oncological and functional outcomes.

## Introduction

**Figure 1:**
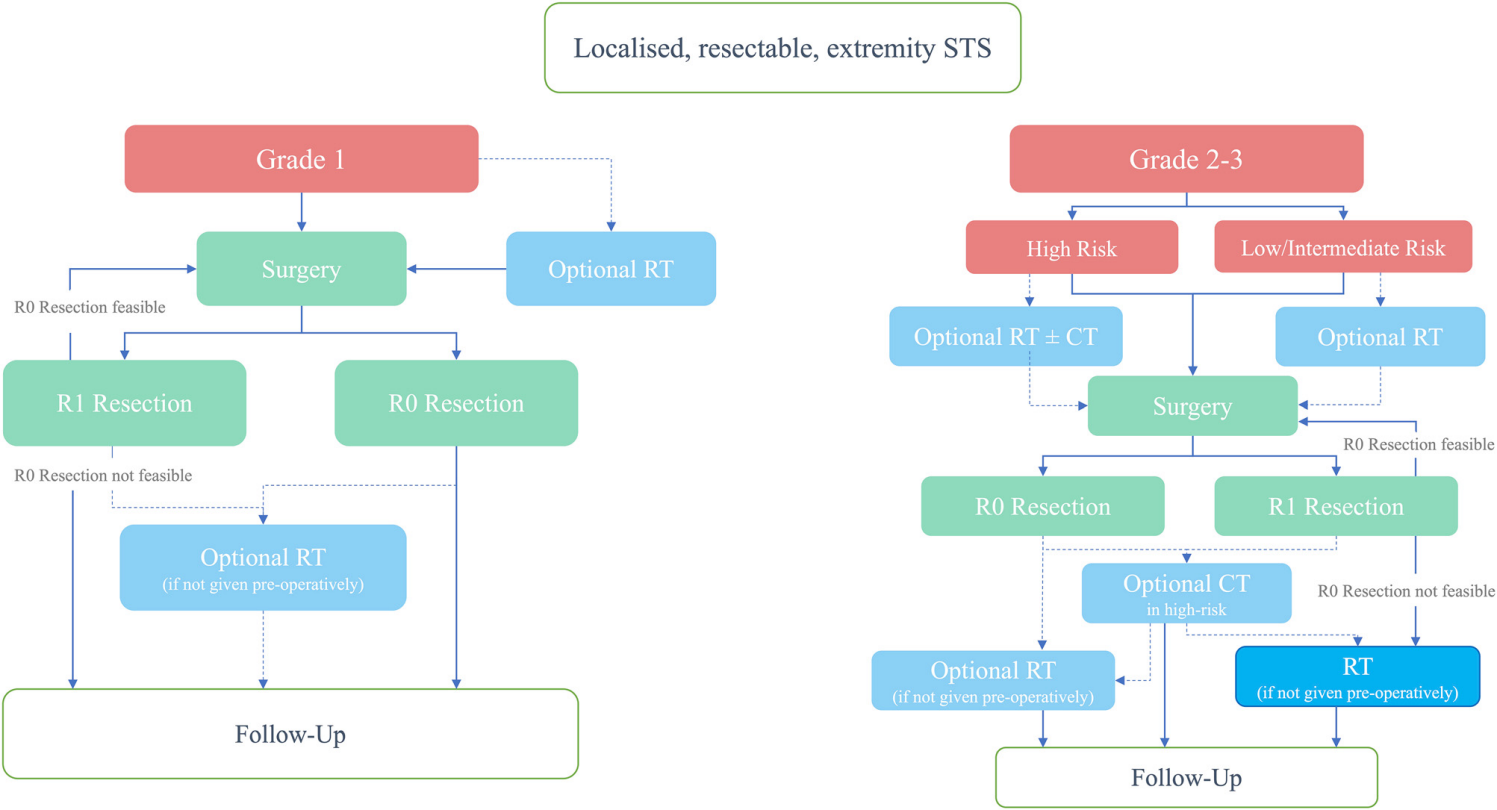
Clinical management of localized, resectable extremity soft tissue sarcomas according to Ref. [1]. Depending on histological grading, the management of G1 sarcomas is differentiated from that of G2/3 sarcomas. From histological grade G2 onwards, a risk assessment should be performed to classify patients into high-vs. low- or intermediate-risk categories. Neoadjuvant or adjuvant radiotherapy (RT) is generally recommended from G2, but may also be considered in selected G1 tumors depending on anatomical location, tumor size, histological subtype, and the anticipated consequences of local recurrence. Chemotherapy (CT) is not considered standard treatment; it is only considered in a limited number of chemosensitive sarcoma subtypes or in selected high-risk constellations. R0=microscopically clear margins, R1=incomplete resection with microscopically positive margins.

The management of soft tissue sarcomas is challenging due to their rarity and heterogeneity and requires a high level of expertise. In the treatment of patients with STS, interdisciplinary collaboration forms the basis of guideline-compliant therapy. The 2021 ESMO-EURACAN-GENTURIS guideline recommends wide excision with histologically negative margins, complemented by radiation and, when appropriate, systemic therapy to maximize local control and overall survival (see Figure 1) [1]. Plastic-reconstructive surgery underpins this strategy: by guaranteeing durable soft-tissue coverage, it expands the surgeon’s freedom to perform radical resections and drives contemporary limb-salvage rates above 90 %. This not only ensures oncological safety, but also significantly improves the quality of life. Although surgical resection is the basis for resectable extremity sarcomas [2], it is crucial for the best possible treatment that interdisciplinary decision-making begins early in the treatment process [3]. Affected patients should immediately be referred to a sarcoma center and treatment decisions should be made in an interdisciplinary tumor conference.

Using the example of a six-year-old girl with advanced epithelioid sarcoma of the hand, for whom amputation was initially recommended, the following demonstrates how an interdisciplinary therapeutic approach – utilizing neoadjuvant radiotherapy, plastic and reconstructive surgery, and oncological expertise – enabled a limb-preserving treatment approach. This case study is intended to illustrate the potential, challenges, and limitations of modern interdisciplinary therapy.

## Therapeutic approaches and interdisciplinary management

### Surgery

#### Limb preservation versus amputation

Limb-sparing surgery with adequate surgical margin combined with reconstructive surgery has replaced amputation as the principal treatment for extremity STS [4]. Achieving adequate surgical margins has been the holy grail of extremity STS surgery and a measure of successful treatment. Adequate surgical margins are generally regarded to translate directly into improved survival by reducing the rates of local recurrence and subsequent risk of metastases [5]. However, attempts to achieve appropriate margins may result in increased morbidity and larger functional deficits. Historically, before reliable flap surgery was established, surgeons often compromised excisional width to achieve primary closure, accepting higher local-recurrence risks. The advent of local perforator, regional, and free-flap techniques permits a “resection-first” philosophy: Defects can afterwards be covered with well-vascularized tissue. Penna et al. reported that free-flap availability increased R0 resection to 92 % and reduced secondary wound-breakdown to 6 % [6].

Although the principle of surgical excision is to obtain a wide resection margin to prevent local recurrence, the size of the resection margin and whether a wide surgical margin with ensuing severe functional disability is warranted are controversial. Our own studies suggest that the width of negative margins seemed to be not relevant [7]. Moreover, adjuvant radiotherapy had a similarly strong impact on local control as well as survival which may be due to the high proportion of high-grade tumors in most studies. With respect to the currently available data from the present and negative margins involving radiotherapy to ensure optimal local control and survival [8].

Large registry analyses confirm that limb-salvage surgery combined with adjuvant radiotherapy yields disease-specific survival equivalent to primary amputation, provided margins are clear [1]. Functional and psychosocial outcomes, however, are markedly superior after limb-salvage surgery. Comparing the functional results of limb salvage and ablative surgeries for STS of the lower extremity using the American Musculoskeletal Tumor Society functional rating system, significantly better functional outcomes were seen in the salvage group of 77 % compared to 60 % in the amputation group [9]. A 2024 MD Anderson cohort of 302 lower-extremity STS patients showed an overall complication rate of 12.5 % in the free-flap subset vs. 42 % after simpler closures, with surgical-site infection falling from 16 to 2.5 % [10]. By minimizing wound morbidity, plastic surgery shortens the delay to adjuvant therapy – an independent prognostic factor for distant-metastasis-free survival.

Functional preservation describes the extent to which physical and psychosocial abilities are maintained after treatment. It can be evaluated using both subjective reports and objective assessments. Patient-reported outcomes include instruments like the Toronto Extremity Salvage Score (TESS) [11] or general health-related quality of life questionnaires like the SF-36. Clinician-reported, objective assessments consist of the Musculoskeletal Tumor Society Score (MSTS) [12], region-specific questionnaires as the Disabilities of the Shoulder and Hand (DASH), as well as performance measures like grip strength and range of motion testing. By combining subjective and objective approaches, a differentiated understanding of the limb function after oncological treatments can be achieved.

#### Timing of reconstruction

Whether coverage should be immediate or delayed remains debated. Haldeman et al. reviewed 34 reconstructions (24 immediate, 10 planned-delayed) and found no difference in wound complications, re-operation rates, or total hospital charges. The study supports tailoring timing to intra-operative realities and theatre logistics. Clinically, however, when neoadjuvant radiotherapy is used, a protocol associated with 30–35 % major wound-complication rates [13]. Immediate microvascular tissue transfer appears protective, replacing irradiated integument with robust, perfused flap tissue.

#### Choice of reconstructive strategy

**Local and regional perforator flaps.** For subfascial tumors of the upper limb and distal leg, fasciocutaneous perforator flaps supply thin, pliable coverage with negligible donor-site morbidity.

**Free flaps.** The anterolateral-thigh (ALT), latissimus-dorsi, and deep-inferior-epigastric-perforator (DIEP) flaps dominate current practice because their pedicles are long, caliber-matched, and tolerant of postoperative radiotherapy. In Penna’s review, flap survival was 96.8 % and overall limb-salvage 94.5 % [6]. When segmental bone resection is inevitable, vascularised osteocutaneous fibula grafts provide immediate structural stability and revascularized bone, allowing weight bearing earlier than endoprosthetic alternatives.

**Functional muscle transfers.** Compartmental resections may sacrifice critical motor units. Abe et al. demonstrated that innervated free-muscle transplantation increased mean Musculoskeletal Tumor Society scores from 76.7 % at 12 months to 83.7 % at 24 months, underscoring the time-dependent nature of re-education [14].

**Nerve and tendon reconstruction.** When long sensory or mixed nerves are excised, cable autografts or processed allografts up to 6–8 cm restore continuity. For key motor deficits, tendon transfers (e.g., tibialis-posterior to dorsiflexors) can augment muscle flaps, preserving proprioception and power.

#### Interaction with radiotherapy and systemic therapy

Meta-analyses show that microvascular reconstruction is safe after both neoadjuvant and adjuvant radiation, without increased flap-loss or systemic complication rates [15]. Well-vascularised tissue beds appear to tolerate postoperative radiation better than native irradiated skin and reduce fibrosis-related contracture. Emerging hypofractionated protocols (e.g., 5 × 5 Gy) may further compress treatment timelines. Therefore, prospective plastic-surgical outcome data are awaited.

#### Complication profile and risk mitigation

Predictors of wound breakdown include lower-extremity location, obesity, diabetes, and pre-operative radiation. Tried strategies to offset risk are meticulous lymphostasis, closed-suction drainage, and routine dual-venous anastomosis for free flaps. Intra-operative indocyanine-green angiography and continuous Doppler monitoring reduce partial-flap necrosis by enabling immediate revision of marginally perfused zones [16], [17], [18], [19].

### Radiotherapy

Radiotherapy (RT) is an essential component in the multimodal treatment of STS, especially for patients presenting with high-risk features, including large tumor size, deep anatomical location or high grade. The primary objectives of RT are to enhance local disease control, preserve function, and lower the risk of recurrence [1], 20], 21].

Neoadjuvant RT is recommended for resectable, high-risk STS, as it enables a smaller target volume, a reduced total dose (typically 50–50.4 Gy in 1.8–2 Gy fractions over 5–6 weeks) and potentially better functional outcomes, although it is associated with an increased risk of wound complications [22]. Adjuvant RT is indicated when adverse pathological findings are identified postoperatively, such as positive surgical margins or unexpected high-risk features and is usually delivered at a higher total dose (60–66 Gy). Severe side effects, including edema, restricted joint mobility, and subcutaneous fibrosis are more common with postoperative RT compared to the neoadjuvant approach. Both strategies achieve comparable local control rates. However, the choice of timing depends on tumor location, resectability, and individual patient factors. Analyses of large datasets suggest that preoperative RT may yield superior oncological outcomes compared to adjuvant RT. Nonetheless, most available data pertain to soft tissue sarcomas larger than 5 cm and of high grade. Therefore, preoperative RT should be considered and discussed for these tumors [23], 24].

The addition of chemotherapy to RT, whether in the neoadjuvant or adjuvant setting, is reserved for selected cases with particularly high-risk features or marginal resectability and is not standard across all STS subtypes. The combination of anthracycline and ifosfamide is most frequently employed in chemotherapy-sensitive histologies, including synovial sarcoma and leiomyosarcoma, while less intensive regimens may be appropriate for frail patients. Concurrent chemoradiation has proven feasibility and does not significantly increase toxicity compared to sequential approaches. However, robust evidence for a survival benefit remains limited. For very large or high-grade STS, neoadjuvant and multimodal therapies should be discussed in an interdisciplinary context. There is evidence that patients receiving simultaneous chemoradiation (with or without hyperthermia) in addition to surgery experience significantly improved oncological outcomes [25]. Recent studies also indicate potential synergies between RT and immunotherapeutic agents, such as pembrolizumab, in the neoadjuvant setting [26].

Modern RT techniques, including intensity-modulated radiotherapy (IMRT), volumetric-modulated arc therapy (VMAT) and image-guided radiotherapy (IGRT) are preferred due to their ability to deliver highly conformal doses while sparing adjacent healthy tissue, thereby reducing late toxicities like fibrosis, lymphedema and bone fractures [27]. Stereotactic body radiotherapy (SBRT) and brachytherapy are options for select situations, for instance, oligometastatic disease or local recurrences [28]. The use of advanced planning and image guidance is strongly recommended to optimize outcomes and minimize complications.

Particle therapy, encompassing proton and carbon ion RT, provides dosimetric advantages via the Bragg peak effect, allowing for maximal tumor dosing with minimal exposure to surrounding tissues. This approach is particularly advantageous for tumors in anatomically challenging regions (e.g., skull base, spine, pelvis) or in pediatric and young adult patients, where tissue preservation is paramount. Proton therapy is associated with lower rates of late toxicity and improved functional outcomes in selected cases. Carbon ion therapy may offer additional biological effectiveness for radioresistant or unresectable tumors, although its application is currently limited to specialized centers and clinical trials [29].

In summary, radiotherapy remains an indispensable element of STS management, particularly in cases with a high risk of local recurrence. State-of-the-art techniques such as IMRT and IGRT are standard, providing effective treatment with reduced toxicity. Proton and heavy ion therapies offer further benefits in selected scenarios, especially for complex anatomical sites or certain subtypes. All treatment decisions should be made collaboratively within an interdisciplinary team and in accordance with established guidelines.

### Systemic therapy

Apart from surgical treatment and radiation therapy, systemic approaches play an increasing role in a multidisciplinary context of the treatment of soft tissue sarcoma. To this end, the exact histological classification of the tumor is crucial as treatment approaches vary among different subtypes of sarcoma. While in the past, post-operative, adjuvant treatment after surgery has been a treatment standard of care for a number of entities depending on certain specific criteria such as grading or size, more and more preoperative, neoadjuvant approaches have been introduced through clinical trials and regular cancer care.

For neoadjuvant treatment approaches, those tumors with risk factors (G2 or G3 differentiation, above 5 cm in size, unfavorable localisation) are eligible for anthracyclin-based chemotherapy, often combined with ifosfamide [30], 31]. The aim of this treatment is downsizing of the primary tumor itself, facilitating operative resection, but also to treat not-visible micrometastases which may be present at initial diagnosis. Approaches to vary the chemotherapy according to histological subtypes in high-risk patients such as gemcitabin/docetaxel for undifferentiated pleomorphic sarcoma, trabectedin for myxoid liposarcoma, high-dose ifosfamide for synovial sarcoma, etoposide/ifosfamide for malignant peripher nerve sheath tumor (MPNST) or gemcitabin/dacarbazin for leiomyosarcoma have unfortunately failed compared to the standard approach with anthracyclins and ifosfamide. Therefore, this combination still remains the standard of care in neoadjuvant treatment [32].

The same criteria apply to adjuvant treatment approaches after tumor resection. Tumors below 5 cm in size or with G1 differentiation are not suitable for adjuvant systemic therapy. For G3 differentiation, an adjuvant chemotherapy can be discussed dependent on the risk profile above (grading and size). However, the decision to add adjuvant systemic treatment is still considered on an individual patient basis [33]. Certain subtypes mainly present in children or younger adults, like Ewing sarcoma or rhabdomyosarcoma, are not discussed in this context, as they are to be treated within multimodal intensive (pediatric) treatment protocols.

In the situation of non-resectable metastases, the histological differentiation becomes far more important as there are systemic treatment options dependent on the subtype of sarcoma. After failure of anthracyclin-based therapy, trabectedin is approved internationally, having been investigated mainly in liposarcomas and leiomyosarcomas, but not limited to these subtypes. For liposarcomas, eribulin is also approved as a 2nd line therapeutic option. DTIC can also be used generally as a 2nd- or 3rd-line option in soft tissue sarcomas. Other cytotoxic drugs, like gemcitabin or docetaxel, are sometimes used in further lines as mono- or combination therapy with dacarbazin. They are subject of current guideline recommendations, however not officially approved for these entities. Angiosarcomas have a high sensitivity for taxanes, underlining the importance of exact histological diagnosis.

Pazopanib is a multikinase inhibitor targeting mainly VEGFR, PDGFR, c-kit and FGFR. It is used in soft tissue sarcomas (apart from liposarcomas) in those patients progressing within a year from adjuvant or neoadjuvant therapy or in 2nd line of metastatic disease. Imatinib, another tyrosine kinase inhibitor which has been developed for Philadelphia positive CML is used and shows high activity in GIST tumours which are also not part of this overview. Imatinib may be applied in relapsed or non-resectable dermatofibrosarcoma protuberans, targeting, among others, c-kit receptors.

There is no clinical benefit in treatment intensification with high-dose therapies (with or without stem cell support) in the metastatic setting. In fact, in the metastastic setting combination treatment with anthracyclines and ifosfamide are not superior to anthracyclines alone. Immunotherapy such as checkpoint inhibition is currently used in many tumor types, with major effects especially in tumors hardly responding to standard chemotherapy, like malignant melanoma or renal cell cancer. However, its effect as a monotherapy in sarcoma treatment is limited, therefore it is so far not approved for sarcoma treatment in the EU. Those patients bearing tumors with mismatch repair deficiency or MSI-H status, however, may be candidates for checkpoint inhibition as an individual approach. Please note, however, that dMMR status is extremely rare in soft tissue sarcoma. Combinations of immunotherapy with standard chemotherapy are currently subject of clinical trials reflecting a high medical need in these patients. Especially in those patients with good clinical performance status after first- or 2nd-line therapy, further genetic analyses may lead to targeted therapeutic options. In the small subgroup of tumours with NTRK-fusions, entrectinib or larotrectinib can be used.

## Case report: epitheloid sarcoma

A 6-year-old girl with a diagnosis of locally advanced epitheloid sarcoma of the right hand was presented in our clinic to obtain a third opinion. Seven months previously, a tumor resection was performed abroad in the form of a whoops procedure. Subsequently, the tumor showed fast growth and the hand function became increasingly reduced, so the family referred to different sarcoma centers for further diagnostics and treatment.

**Figure 2:**
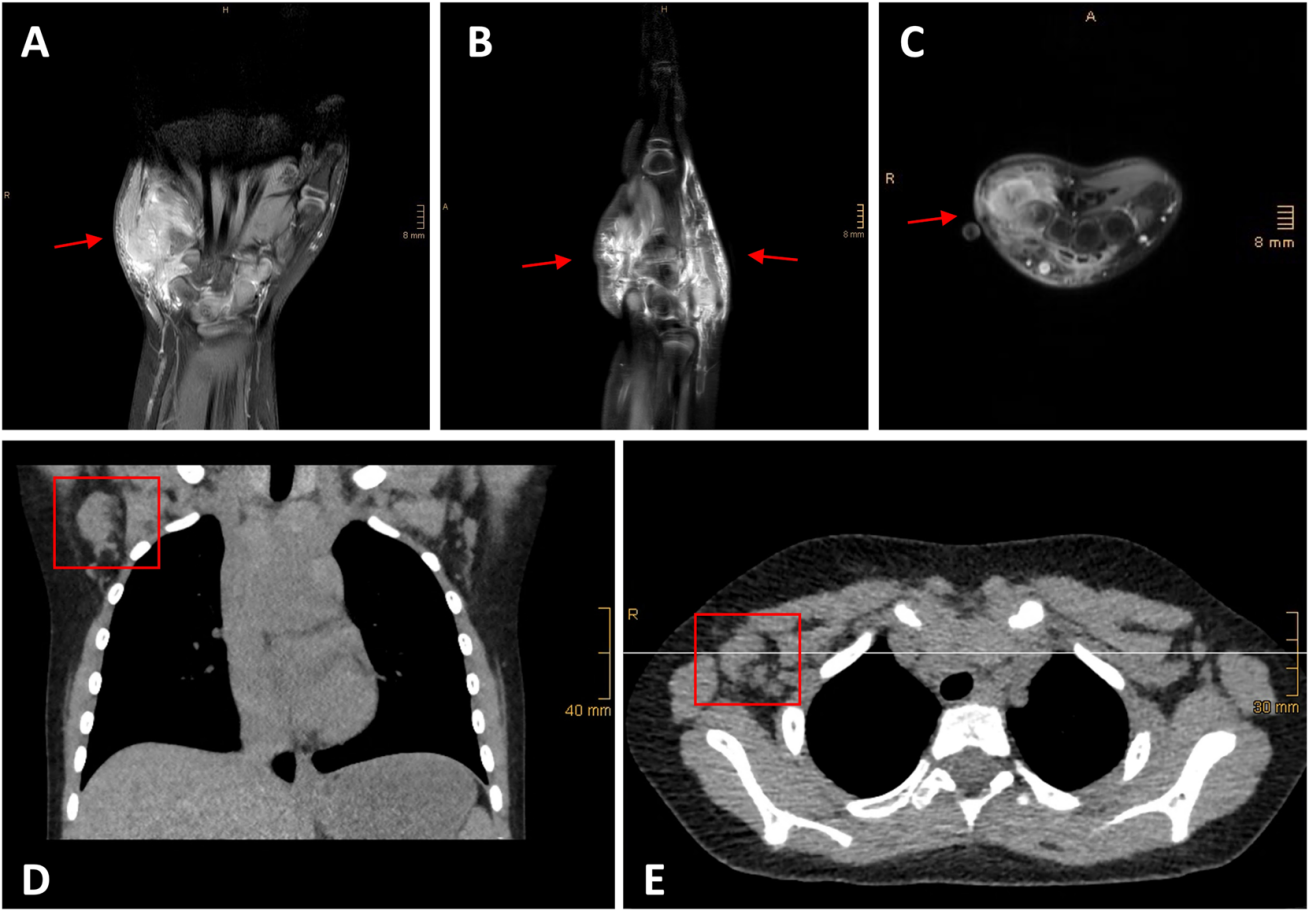
Initial contrast-enhanced MRI pictures of the right hand coronal (A), sagittal (B) and axial (C) view. Computed tomography (CT) scan of the thorax revealed an enlarged lymph node in the right axilla (D coronal and E axial view).

Initial contrast-enhanced MRI revealed a large soft tissue tumor which infiltrated the fifth metacarpal and extended semicircular from the dorsal aspect of the hand, through the carpus – along the capitate and the base of the fourth metacarpal – and further along the ulnar border of the hand, infiltrating the triangular fibrocartilage complex (TFCC) toward the palmar side. The staging PET-CT showed a metabolically positive axillary lymph node ipsilateral without other signs of distant metastasis (see Figure 2).

The case was demonstrated and discussed in multidisciplinary tumor boards involving pediatric oncology, radiation therapy and plastic surgery. Due to the size and location of the tumor, an amputation at the level of the distal forearm was proposed. The patient and the relatives preferred limb preserving treatments, so isolated limb perfusion was considered as an alternative salvage option, but refused given the potential side effects. Chemotherapy was not recommended due to an expected poor response rate. Neoadjuvant radiotherapy in volumetric modulated arc technique (VMAT) was conducted with a total dose of 45 Gy.

**Figure 3:**
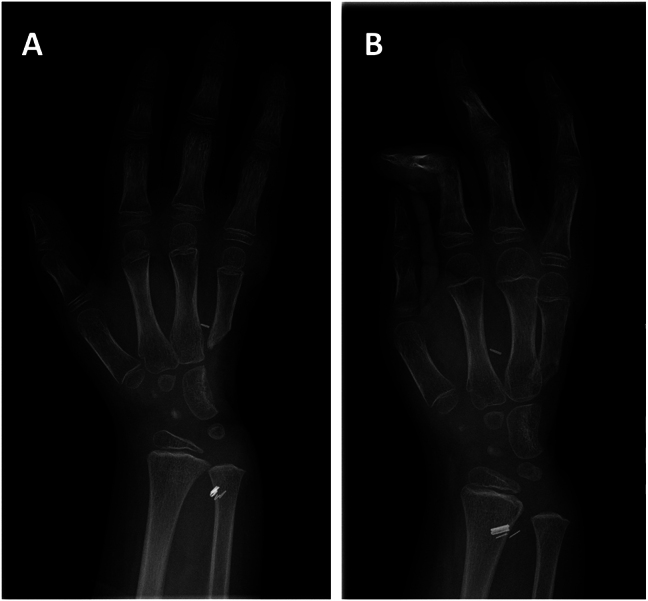
Postoperative radiography of the hand after tumor resection and primary closure; (A) anteroposterior view, (B) lateral view.

**Figure 4:**
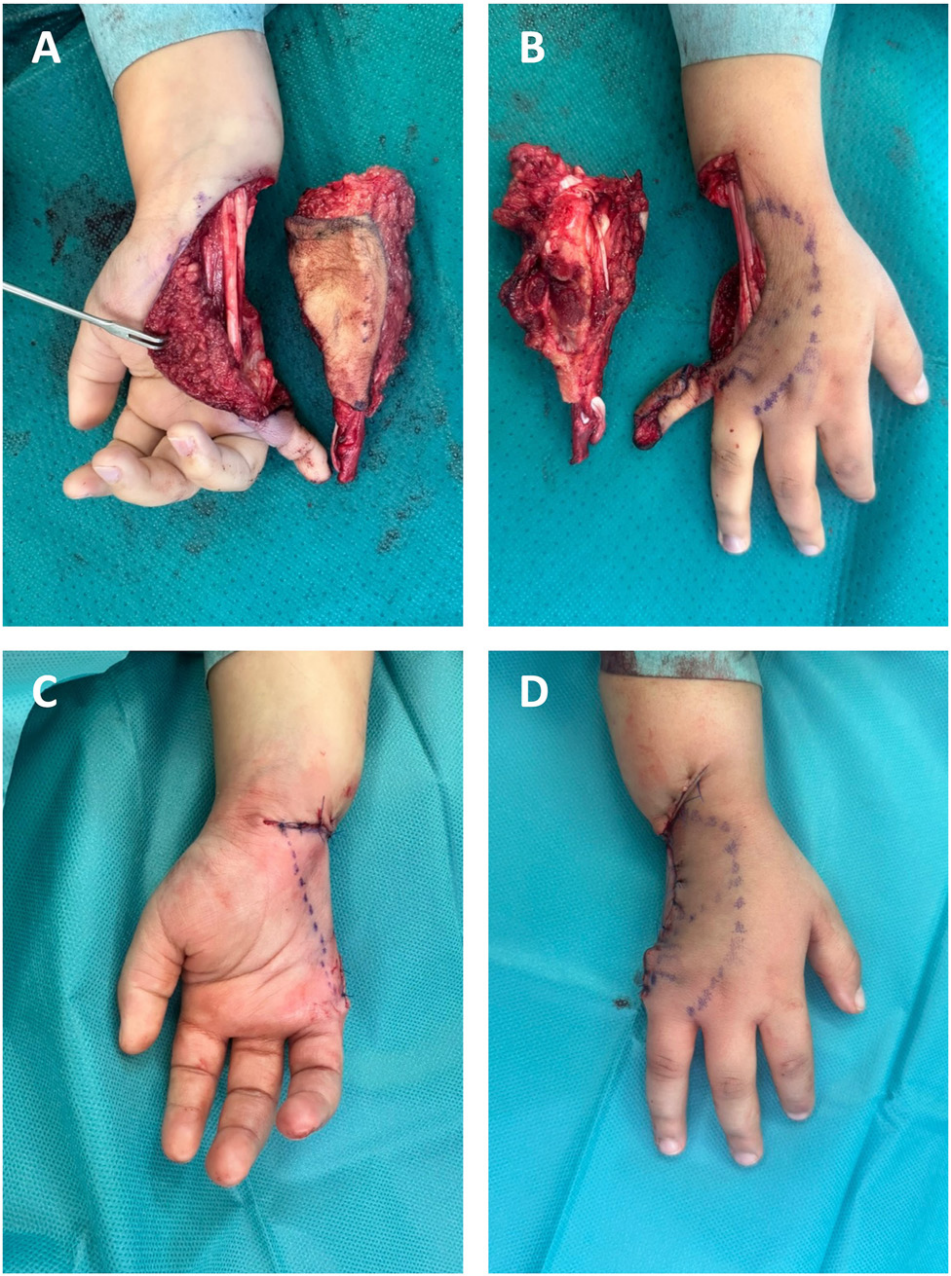
Perioperative (A,B) und postoperative (C,D) clinical presentation of the hand.

After radiotherapy, MRI und CT diagnostic showed no change of the positive lymph node and the tumor considerably decreased in size. Concerning the lymph node, a watch-and-wait strategy with frequent follow-up assessments was advised. With regard to the tumor, surgical treatment was recommended and included excision of the biopsy scar, resection of the fifth finger and metacarpal, resection of the base of the fourth metacarpal, removal of the hamate, triquetrum, pisiform and the ulnar border of the capitate as well as the TFCC and ulnar artery and nerve Figure 3. Primary closure was achieved, so no free flap reconstruction was required, contrary to previous expectations (see Figure 4). Histopathological examination showed no viable tumor cells (ypT0) and clear resection margins (R0) confirming the effectiveness of radiotherapy and the interdisciplinary approach. Although a postoperative wound healing disorder occured after irradiation of the tissue, no further surgical treatment was required (Figure 4).

**Figure 5:**
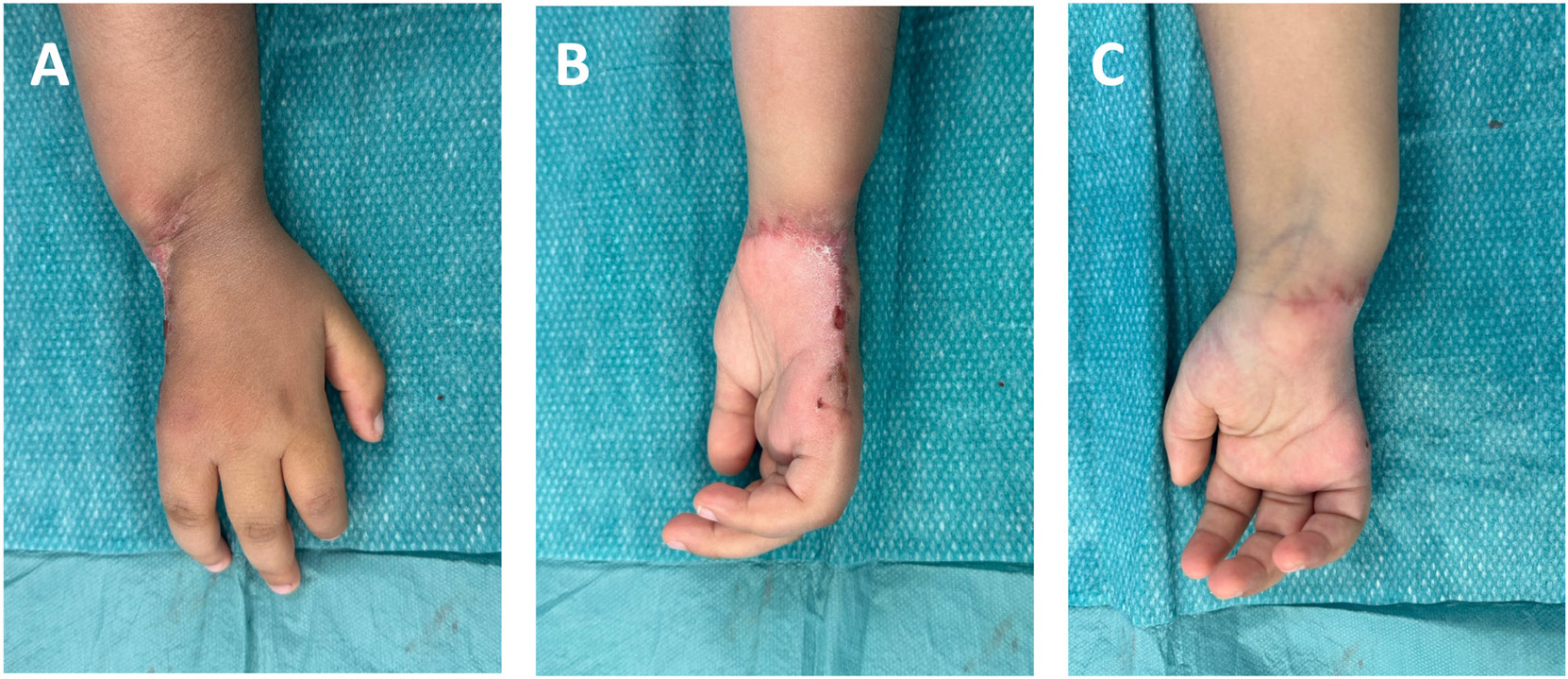
Healed postoperative results of the right hand in a relaxed resting position from dorsal (A), ulnar (B) and palmar (C) view.

At 3-month follow up, the patient showed a good hand function with only a minimal residual extension deficit of the ring finger with a fingernail-to-table distance of 1.5 cm during attempted active extension (Figure 5). In addition to intensive physiotherapy and occupational therapy, close radiological follow-up examinations were carried out using contrast-enhanced MRI and axillary lymph node sonography. The enlarged axillary lymph node was initially interpreted as most likely reactive, related to prior radiation and surgical therapies and the temporary wound healing disorder, and not necessarily suspicious for metastasis. However, due to the lack of size regression after a three-month observation period, surgical resection was recommended in the tumor board. Subsequently a lymph node extirpation (size 2.6 × 2 × 1.6 cm) was performed (R0), and histopathology revealed a metastasis of the epithelioid sarcoma. The patient continues to receive close follow-up examinations.

## Discussion

Early plastic and reconstructive assessment is recommended, especially in pediatric sarcomas at functionally highly relevant sites (e.g., the hand). This case illustrates how inadequate (surgical) therapies can complicate further treatment, but also how interdisciplinary treatment and modern plastic surgery can ensure radical oncological outcomes without compromising function.

### Importance of interdisciplinary tumor board and specialized tumor centers

As the case illustrates, the initial assessment of the extent of surgery can be reassessed in an interdisciplinary context and enable limb-preserving treatments. In addition to the primary treatment disciplines (surgery, oncology, radiotherapy), other disciplines such as radiology, pathology, and human genetics must be involved, particularly in the context of diagnostics and treatment planning [34]. Furthermore, in many cases, depending on tumor location and spread, the development of a joint treatment plan is required within the scope of surgical treatment [35]. The collaboration also enables inclusion in clinical trials. To develop an individualized, guideline-compliant treatment plan for each patient, it is essential that the patient is presented at an interdisciplinary oncology conference [34]. This not only defines the treatment concept in general (e.g. primary surgical resection), but also – if necessary – the exact procedure (e.g. metastasectomy surgery, amputation, etc.) [36].

**Histological diagnosis.** High expertise is required even during diagnosis, as the reproducibility of an STS diagnosis is relatively poor among pathologists unfamiliar with these lesions [37].

**Surgical outcome.** Regarding surgical resection, it has been shown that patients operated in centers with high case volumes achieved a significantly higher rate of microscopically negative margins [38]. The survival rate was also higher in patients who underwent surgery in specialized tumor centers than in patients who underwent surgery in hospitals with less experience in sarcoma surgery: 2-year survival rate 87 vs. 84 %, 5-year survival rate 73 vs. 65 %, 10-year survival rate 58 vs. 53 % [39]. Unplanned excisions (whoops surgeries) are often performed for smaller masses and lead to R1 resections in 55 % of cases with an associated reduced probability of survival, more frequent metastasis and higher amputation rates for sarcomas of the extremities (18 % for R1 vs. 1.8 % after R0 resection) [40].

In addition to the extensive experience of the medical treatment team, patients in specialized sarcoma centers can also benefit from the high level of expertise of the nursing staff, support from specialized physiotherapists and occupational therapists, as well as additional services such as by oncological psychotherapists.

With regard to the two-stage approach to lymph node resection in this case, several aspects need discussion. Initially, the lymph node was not considered suspicious for metastasis, despite an elevated risk of lymphatic spread in epithelioid sarcoma reported in different studies (12.4–16.7 %) [41], 42]. During the treatment, additional plausible explanations for reactive lymph node enlargement were present, including radiotherapy, surgery, and the subsequent wound healing disorder. Only after there was no size regression, an (excisional) biopsy was recommended. Importantly, in pediatric patients the tendency to minimize repeated surgical interventions may sometimes contribute to underestimation of disease extent, particularly in centers lacking dedicated pediatric surgical expertise.

Before free flap reconstruction became widespread, limb-sparing approaches tended to be associated with a higher risk of „undertreatment“ [6]. It has since been demonstrated that in specialized centers, where plastic surgeons with expertise in sarcoma surgery and microsurgical reconstruction are available, the rates of successful limb preservation are significantly higher compared to non-specialized institutions [43]. As long as the resection margins are clear and (neo-)adjuvant radiotherapy is performed, preserving the limb does not compromise oncological safety.

### Limitations of therapy

The multimodal treatment of sarcoma patients has advanced in recent years across virtually all disciplines involved in treatment. In addition to established therapeutic approaches for limb preservation and the establishment of plastic-reconstructive procedures for soft tissue reconstruction, several adjuvant and neoadjuvant treatment concepts have been established in clinical trials. Nevertheless, some significant limitations remain. The role of systemic therapy remains unclear in many subtypes and is often characterized by low evidence. If isolated limb perfusion, radiotherapy or neoadjuvant systemic therapy fails, amputation remains the last option for a curative approach in some cases [44], especially when functional structures are infiltrated. Depending on the extent of distant metastases, palliative treatment may be the only option in some cases. Furthermore, it must be considered that even with histologically confirmed negative resection margins, a risk of recurrence remains. This risk can be reduced by adjuvant therapy but can never be eliminated completely.

### Future directions

Future treatment of soft tissue sarcomas shows promising developments: Molecular analyses and next-generation sequencing are enabling targeted therapies, even for previously refractory tumor entities [45]. The integration of artificial intelligence into radiological diagnostics and follow-up care can optimize the safety of therapies [46]. Surgical future therapy options include:

Super-microsurgical lymphovenous anastomoses to prevent radiation-induced lympho-edema; three-dimensional printed, patient-specific titanium implants anastomosed with soft-tissue flaps to broaden indications for joint-sparing resections and targeted muscle reinnervation and regenerative peripheral-nerve interfaces, originally developed for amputees, as adjuncts to flap neurorrhaphy to enhance electromyographic prosthesis control and reduce neuroma pain.

Another key lies in centralized care and quality assurance. Initiatives such as the German Sarcoma Foundation, the European Society for Medical Oncology (ESMO), the European Reference Network for Rare Adult Solid Cancers (EURACAN) and the European Reference Network for Genetic Tumor Risk Syndromes (GENTURIS) promote the exchange of data and expertise and ensure the continuous development of therapy guidelines. In the future, all STS patients should be referred to a specialized center and treated in a multidisciplinary setting – regardless of age, place of residence, or tumor stage.

## Conclusions

Interdisciplinary therapy of patients with soft tissue tumors is the basis for adequate treatment. In addition to initial planning within the framework of an interdisciplinary oncology conference, multimodal treatment should be carried out in a specialized center to guarantee the best possible and comprehensive treatment. Plastic-reconstructive surgery is indispensable to contemporary STS therapy. By enabling radical oncological clearance, preserving limbs, and restoring complex anatomy, it transforms limb-salvage from theoretical possibility to reproducible standard. Seamless integration of plastic surgeons remains the cornerstone of optimal oncological and functional outcomes. The presented case of a pediatric epithelioid sarcoma exemplifies how close interdisciplinary cooperation can enable individual, function-preserving treatment options to be implemented even in complex situations.
